# A synopsis of East-Mediterranean *Synaphris* Simon, 1894 (Araneae, Synaphridae) with a description of a new species from Israel

**DOI:** 10.3897/zookeys.82.957

**Published:** 2011-02-23

**Authors:** Yuri M. Marusik, Sergei Zonstein

**Affiliations:** 1 Institute for Biological Problems of the North RAS, Portovaya Str. 18, Magadan, Russia; 2 Department of Zoology, The George S. Wise Faculty of Life Sciences, Tel-Aviv University, 69978 Tel-Aviv, Israel

**Keywords:** Spiders, East Mediterranean, distribution

## Abstract

Three species of Synaphris occurring in the East Mediterranean – Synaphris orientalis Marusik & Lehtinen, 2003, Synaphris lehtineni Marusik, Gnelitsa & Kovblyuk, 2005 and Synaphris letourneuxi (Simon, 1884) – are surveyed; and a new species – Synaphris wunderlichi **sp. n.** – is described from southern Israel on the basis of males. The new species differs from other East- Mediterranean congeners by its smaller size, a smaller lamella with fewer ridges, and a thick palpal femur. Comparative figures are provided for all species from the East Mediterranean.

## Introduction

Synaphridae Wunderlich, 1986 is a small family with three genera and 12 species known from the Mediterranean region (including adjacent territories: the Canary Islands and western Turkmenistan) and Madagascar (cf. Platnick 2011). The most species-rich genus in the family is Synaphris, containing 10 species: eight from the Mediterranean (from the Canary Islands to western Turkmenistan) and two from Madagascar. Its type species, Synaphris letourneuxi (Simon, 1884), was originally described in Grammonota Emerton, 1882, a linyphiid genus. A decade later after the species description a new genus was suggested for it (Simon 1894). This genus was later considered within the Theridiidae and Symphytognathidae, until Wunderlich (1986) placed it in a separate subfamily of Anapidae. The group was given family status by Marusik and Lehtinen (2003). Less than a decade ago, this family was known exclusively from the south-western Palaearctic. Recently, Miller (2007) reported this family from Madagascar and described two species of Synaphris and one new monotypic genus, Africepheia Miller, 2007. This finding suggests that the Synaphridae are more widespread than previously assumed and probably also occur in eastern Africa.

While studying material collected in Israel by pitfall traps we identified over two dozen specimens belonging to Synaphris and initially thought they might be Synaphris letourneuxi, the species described from Egypt and known only from the male holotype. A detailed examination of our specimens, as well as their comparison with the literature and all the available material, has revealed them to belong to an unknown species. This study surveys all the species currently known from the East- Mediterranean region and describes a new species.

## Material and methods

Digital photographs of general appearance and copulatory organs were taken using an Olympus SZX16 stereomicroscope with an Olympus E-520 camera and prepared using the CombineZP software. These photographs were taken in alcohol, in dishes with paraffin at the bottom. Different-sized hollows were made at the bottom to maintain the specimens in the desired position. Scanning electron photos were taken using the SEM JEOL JSM-5200 scanning microscope at the Zoological Museum, University of Turku. All measurements are in mm. Type material will be deposited at the Department of Zoology, Tel-Aviv University (TAU), the National Spider Collection at the Hebrew University, Jerusalem (HUJ), Göteborgs Naturhistoriska Museum (GNM) and the Zoological Museum, University of Moscow (ZMMU). The terminology follows Marusik and Lehtinen (2003). Only one abbreviation has been used on the figures: *La* – lamella. All measurements are in mm.

## Taxonomic survey

To date, three species of Synaphris have been known from the East-Mediterranean region (east of 20°E) (cf. Platnick 2011). All of them are known from type localities only. A synopsis of these species including the new one is given below.

### Synaphris lehtineni

Marusik, Gnelitsa & Kovblyuk, 2005

Figs 12, 15 16

Synaphris lehtineni Marusik et al. 2005: 125, f. 1–4, 6–14, 18–31 (♂♀).

#### Comments.

This species was described on the basis of 30 specimens collected from a single Crimean locality (Marusik et al. 2005). After the species was described, repeated attempts to recollect it from the type locality have been unsuccessful (Kovblyuk pers. comm.). This may indicate that its population density can fluctuate significantly. Although not recollected from the type locality, it was found in one more locality on the south-eastern coast of the Crimean peninsula (Kovblyuk et al. 2008). Here we provide only comparative figures that enable its discrimination from other East-Mediterranean species*. S. lehtineni* is the northernmost species of the genus. All specimens were found under stones in the sub-Mediterranean Quercus-Pistacia-Abies-Juniperus forest, on small sheet-webs (Kovblyuk pers. comm.).

### Synaphris letourneuxi

(Simon, 1884)

Figs 21–22

Grammonota letourneuxi Simon 1884: 599 (♂).
Synaphris letourneuxi : Simon 1894: 589.
Synaphris letourneuxi : Levi and Levi 1962: 64, f. 311 (♂).
Synaphris letourneuxi : Brignoli 1970: 1407, f. 7–10 (♂).
Synaphris letourneuxi Wunderlich 1980: 259, f. 15–16 (♂).
Synaphris letourneuxi : Wunderlich 1987: 137, f. 363 (♂).

#### Comments.

This is the type species of the genus. The species remains known from the male holotype only, collected in Aswan (=Assuan, Egypt). Although it has been redescribed several times, details of its male palp remain unknown. Neither lamella, nor the embolus basis, the course of the seminal duct or position of the cymbial furrow have been depicted or verbally described.

### Synaphris orientalis

Marusik & Lehtinen, 2003

Figs 13 17

Synaphris orientalis  Marusik & Lehtinen 2003: 150, f. 1–24 (♂).
Synaphris orientalis : Marusik et al. 2005: 128, f. 5, 15–17, 32 (♂).

#### Comments

Like the type species, Synaphris orientalis remains known from the male holotype only, collected in western Turkmenistan. Despite this, the species was studied by means of scanning electron microscope and properly described by Marusik and Lehtinen (2003).

### Synaphris wunderlichi sp. n.

urn:lsid:zoobank.org:act:90027054-25C3-43EB-9C22-462F3640BCD0

Figs 1 11, 14 18–20 

#### Material

Holotype ♂ (TAU) and paratypes 29♂♂ (HUJ, TAU, GTM & ZMMU) ISRAEL: Adulam 8 km SSW Beit-Shemesh, 31°39'N, 34°57'E, 350–400 m, oak maquis (Quercus calliprinos), pitfall traps, 15.04.2003 (U. Columbus & T. Levanony).

#### Note

Although the species was numerous in pitfall traps, the second author (SZ) was unable to find any specimen by hand-picking or sifting the litter.

#### Etymology

The species name is a patronym in honour of our friend and colleague, the noted arachnologist Jörg Wunderlich (Germany), who erected the subfamily Synaphrinae.

#### Diagnosis

The new species can be separated from other East-Mediterranean species, Synaphris orientalis, Synaphris lehtineni and Synaphris letourneuxi, by its smaller size (carapace < 0.5, in all other species longer than 0.5). In addition to size, the new species can be recognized by the relatively smaller lamella (cf. Figs 8, 12–13), with less developed ridges. Number of lamellar ridges in the new species (about 6) is approximately half that of its East-Mediterranean congeners. In addition, Synaphris wunderlichi sp. n. has a relatively shorter and thicker palpal femur (cf. Figs 14–15, 17–19, 22). The new species is most similar in size to Synaphris dalmatensis Wunderlich, 1980, but the Balkan species has relatively longer legs, and unlike other Synaphris species, it has tarsus I shorter than metatarsus I (0.24 and 0.21 respectively). The shape of the lamella in Synaphris dalmatensis is unknown.

#### Description

Male. Total length 0.91–0.96. Carapace: 0.46 long, 0.41 wide, uniformly coloured light brown with three dorsal median setae as in other species. Abdomen oval, lighter than carapace, without pattern.

Leg joint measurements:

**Table:** 

	Femur	Patella & Tibia	Metatarsus	Tarsus	Total
I	0.357	0.400	0.243	0.257	1.257
II	0.357	0.386	0.200	0.243	1.186
III	0.314	0.314	0.200	0.243	1.071
IV	0.386	0.371	0.214	0.257	1.228

**Figures 1–7.**
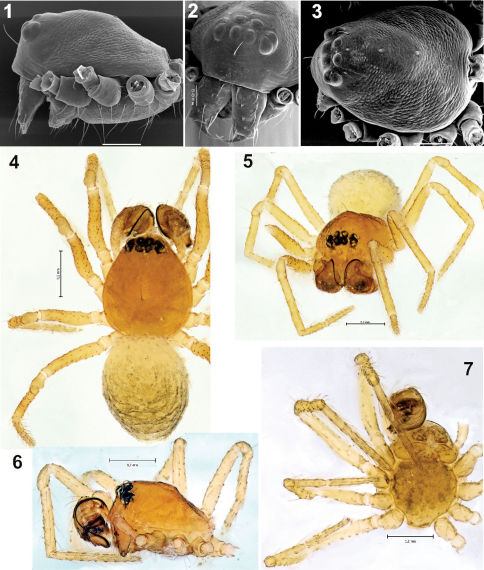
Prosoma and habitus of Synaphris wunderlichi sp. n. **1–3** prosoma with removed legs and palps, lateral, frontal and dorsal **4–5** habitus, dorsal and frontal **6–7** prosoma, lateral and ventral. Scale = 0.1 mm if not otherwise stated.

**Figures 8–15.**
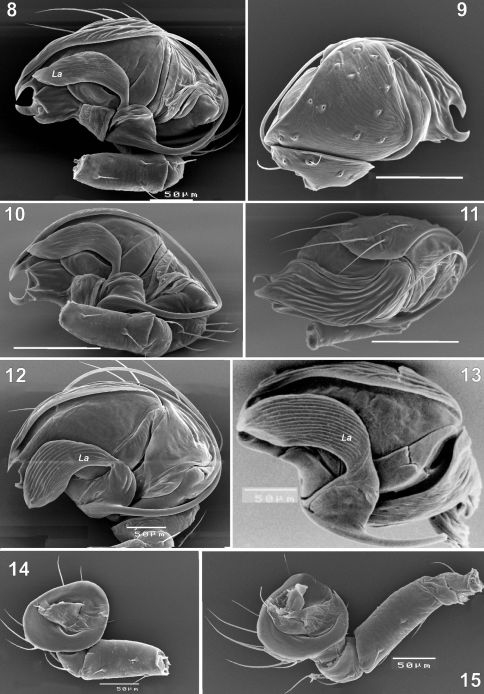
Scanning electron microphotographs of the male palp of Synaphris wunderlichi sp. n. (**8–11, 14**), Synaphris lehtineni (**12, 15**) and Synaphris orientalis (**13**). **8, 12–13** prolateral **9** retrolateral **10** caudal **11** anterior **14–15** palp with removed bulbus showing femur-tibia, anterior. Scale = 0.1 mm if not otherwise stated.

**Figures 16–22.**
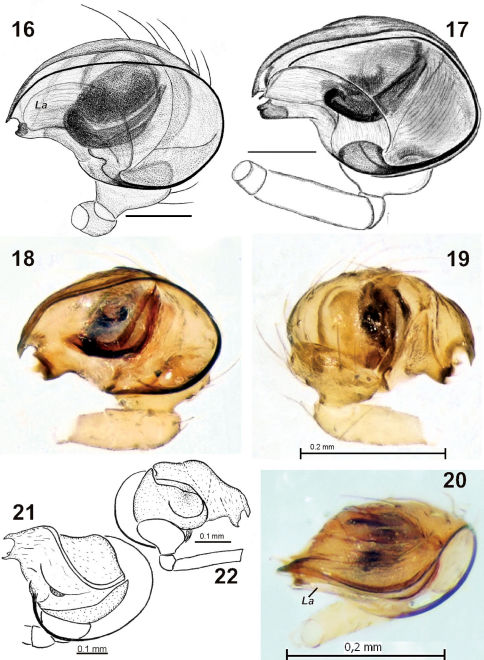
Male palp of Synaphris lehtineni (**16**), Synaphris orientalis (**17**), Synaphris wunderlichi sp. n. (**18–20**) and Synaphris letourneuxi (**21–22**). **16–18** prolateral **19, 22** retrolateral **20** anterior **21** retrolateral-anterior. Scale = 0.1 mm if not otherwise stated. **16–17** after Marusik et al. (2005); **21–22** after Wunderlich (1980).

The palp as in Figs 8–11, 14, 18–20. Femur short and thick; patella small; tibia wide, round and flat (Fig. 14); lamella lanceolate with six longitudinal ridges, lamella invisible in compound microscope in prolateral view, but can be found in terminal view (Fig. 20); seminal duct in the base of embolus is straight (Fig. 18).

**Distribution**. Type locality only.

**Comments**. When we first examined these specimens from Israel, we thought that they might be conspecific with the generotype, Synaphris letourneuxi, described and known from neighbouring Egypt. The type locality of Synaphris letourneuxi, near Aswan (=Assuan), is quite distant from southern Israel. The holotype of Synaphris letourneuxi is 1.28 long, with carapace 0.53 long, distinctly larger than the new species. In addition to differences in the terminal part of the bulbus, Synaphris letourneuxi has a thinner and relatively longer palpal femur (cf. Fig. 22). Unfortunately, the lamella in this species remains unknown, as in all other species described prior to its first observation in 2003.

**Map 1.**
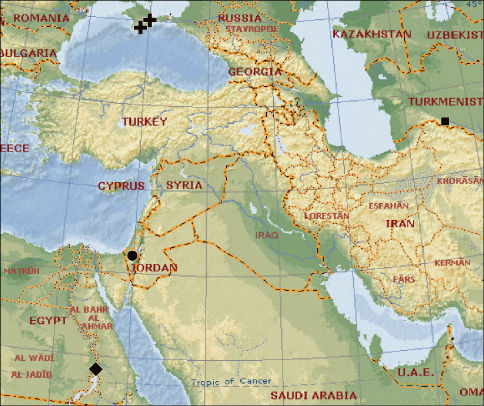
A map showing type localities and distribution of Synaphris letourneuxi (♦), Synaphris lehtineni (+), Synaphris wunderlichi sp. n. (•) and Synaphris orientalis (■).

## Conclusions

All Synaphris species are very similar in general appearance and differ only in details of the terminal part of the bulbus and shape of the lamella; the latter is yet known only in Synaphris wunderlichi sp. n. Females are known in a few species, making it impossible to provide an identification key for the entire genus, or even for the species occurring in the East Mediterranean. Nevertheless, the species living eastward of 20°E can be easily differentiated by their sizes (Table 1)

**Table 1.** Comparison of size of East Mediterranean Synaphris species.

	Total	Carapace length	Leg I	Femur I
Synaphris wunderlichi sp. n.	0.91–0.96	0.46	1.26	0.36
Synaphris orientalis	1.06	0.54	1.4	0.43
Synaphris letourneuxi	1.28	0.53	1.56	0.47
Synaphris lehtineni	0.96–1.09	0.52–0.54	1.29	0.38

Interestingly, all the Synaphris species described from the Palaearctic Region, except for Synaphris lehtineni known from two localities (see Map 1) are known from a single locality, whereas both species from Madagascar were found in several localities, even on the opposite sides of the island. The same holds true for Cepheia longiseta (Simon, 1881), which is known from at least seven separate localities, from south-west Portugal to Switzerland (Lopardo et al. 2007). In the Palaearctic Region all Synaphris species are allopatric, whereas in Madagascar there are four localities in which both Synaphris schlingeri Miller, 2007 and Synaphris toliara Miller, 2007 co-occur (see Miller 2007).

Given that all the Palaearctic species have a very local distribution, it is likely that any new findings may represent a new species. We expect a true species diversity of Synaphris to be at least twofold its presently known one.

## Supplementary Material

XML Treatment for Synaphris lehtineni

XML Treatment for Synaphris letourneuxi

XML Treatment for Synaphris orientalis

XML Treatment for Synaphris wunderlichi
